# The Influence of Serious Games in the Promotion of Healthy Diet and Physical Activity Health: A Systematic Review

**DOI:** 10.3390/nu15061399

**Published:** 2023-03-14

**Authors:** Susana Lamas, Sofia Rebelo, Sofia da Costa, Helena Sousa, Nelson Zagalo, Elisabete Pinto

**Affiliations:** 1 Centro de Estudos de Comunicação e Sociedade, Instituto de Ciências Sociais, Universidade do Minho, Campos de Gualtar, 4710-057 Braga, Portugal; 2 CBQF—Centro de Biotecnologia e Química Fina, Laboratório Associado, Escola Superior de Biotecnologia, Universidade Católica Portuguesa, 1649-023 Porto, Portugal; 3 EPIUnit—Instituto de Saúde Pública, Universidade do Porto, 4099-002 Porto, Portugal; 4 DigiMedia, Universidade de Aveiro, Campus Universitário de Santiago, 3810-193 Aveiro, Portugal

**Keywords:** serious games, nutrition, childhood obesity, real foods

## Abstract

(1) Background: serious games seem to show promising strategies to promote treatment compliance and motivate behavior changes, and some studies have proven to contribute to the literature on serious games. (2) Methods: this systematic review aimed to analyze the effect of serious games in promoting healthy eating behaviors, effectively preventing childhood obesity, and improving physical activity in children. Five electronic bibliographic databases—PubMed, ACM Digital Library, Games for Health Journal, and IEEE Xplore were used to conduct a systematic literature search based on fixed inclusion and exclusion criteria. Peer-reviewed journal articles published between 2003 and 2021 were selected for data extraction. (3) Results: a total of 26 studies were identified, representing 17 games. Half of the studies tested interventions for healthy eating and physical education. Most of the intervention’s games were designed according to specific behavioral change theories, predominantly the social cognitive theory. (4) Conclusions: studies confirmed the potential of serious games for obesity prevention but considering the restrictions encountered, we exhort for novel designs with different theoretical perspectives.

## 1. Introduction

In recent decades, the prevalence of obesity in children has increased significantly, and it is now recognized as a global epidemic [1]. Multiple complex factors are associated with this prevalence [2], namely behavioral factors such as decreased PA and increased consumption of unhealthy food choices [3]. There is growing evidence of the impact that being overweight and obese have on short and long-term health and well-being [4], supports the relevance of their prevention as public health priorities [2,5].

The prevalence of obesity in children has increased dramatically, as evidenced in 2015, when a total of 107.7 million children were classified as obese, corresponding to a worldwide prevalence of childhood overweight and obesity of 23% [6]. Once established, it is difficult to reverse and likely to persist into adult life [7].

Obesity is a multisystem disease characterized by the accumulation of excessive fat in the body [3], already defined by the World Health Organization (WHO) as a condition of abnormality in the content of body fat, or an excess of fatty tissue, which affects or deteriorates the health of an individual [1]. This is associated with the development of chronic non-communicable diseases such as hypertension, type 2 diabetes mellitus, hepatic steatosis, and dyslipidemia in children, which were previously considered to be “adult diseases,” favoring the increased risk of early mortality in adulthood [1].

One of the most frequent behavior patterns among children is to spend too much time in front of a screen (television (TV), computers, tablets). They often do this while they are eating, which causes a greater food intake as well as less energy expenditure (sedentary behavior). This has created a link between childhood obesity and food intake [8], combined with a lack of PA. In addition, commercials and other programs on TV are one of the drivers of this problem, due to the favorable publicity they give to unhealthy foods, which has a negative impact on the relationship between children and food [9]. It was also detected that destructive health behaviors generally prevail throughout the family, so that the possibility of a child becoming an obese adult is as high as 80%. Therefore, it is clear that an obese child will more likely become an obese adult [8].

It is imperative to find new methods to combat this epidemic that involves children from an early age and the family, rather than individual component programs [10]. Serious games make up part of this diversity of strategies and tools to intervene in young age groups due to the predisposition of children to learn, coupled with their attraction to video games. 

Most 10-year-old children spend more than one hour per day playing digital games in Europe and the United States of America [11]. Videogames appeal to both children and their parents (in the USA, 74% of parents play games with their children on a weekly basis at least) [12]. 

Serious games are video games designed to achieve a change of some type while entertaining [13]. In health, they are targeted towards the rehabilitation of patients suffering from various disabilities [14], for the treatment of specific diseases [15], and also for the promotion of healthy lifestyles [16]. In 2013, video games for self-healthcare and wellness (which exclude games for patients and professional training) accounted for 41% of all health games [17]. 

Beyond the revision of serious games for promoting good health-related behaviors [18], some reviews addressed nutrition and obesity prevention. Mack et al. (2017) reviewed video games explicitly targeting nutrition, PA, and obesity for children between 7 and 15 years of age [19]. Baranowski et al. (2019a) made a scoping review of nutrition education and dietary behavior change videogames/or interactive games (as part of human-delivered interventions) [20]. Moreover, Chow et al. (2019) gathered forty-three studies that used video or physical games and gamification (i.e., game-based approaches) to influence children’s (≤12 years old) eating behavior [21]. 

From the above, it is still rather unclear what the contribution of challenging games is to a change in lifestyles, mainly in terms of reducing obesity. However, they do show a clear potential to induce change. Effects are minor [19] and are primarily reported in the short term [21]. Games differ remarkably in their targeted knowledge and behaviors, population, game mechanics, behavioral theories, research designs, and findings [20], hampering the analysis. A more solid research-based game design and evaluation would enable a more in-depth analysis of serious games’ characteristics and outcomes [20] to conclude their effectiveness [16] properly.

While authors such as Baranowski and colleagues (2019a) appeal to the embracing of behavior change procedures in games’ design, trying to change the player’s attitudes, beliefs, risk perceptions, knowledge, or skills in the mediation to better health outcomes [20], others such as Guy, Ratzki-Leewing, and Gwadry-Sridhar (2011) [22] remind us that nutritional expertise and financial resources seem not to be the leading solutions to healthy eating. There is a need for an ecological, multilevel approach to childhood obesity prevention [23].

In this light, games would benefit from acknowledging the kid’s food environment in their narratives (e.g., the pressure of publicity/markets on children’s food choices and the food available in their environment). Likewise, they would benefit from recognizing social stigmas in girls’ sports practices as barriers to PA. This would leverage communication beyond solely depicting information (e.g., the nutritional content of foods, pointing to what is good or bad). Games can propose solutions to barriers or provide extra motivational arguments for behavioral change by acknowledging reality.

Additionally, as far as eating behavior is concerned, there seems to be a distinct lack of games to encourage children’s (sensorial) food exploration. In analyzing a diversity of games, Chow et al. (2019) [21] only found one article that developed a digital experience with natural foods [24]. EducaTableware hopes to help kids enjoy eating disliked foods in dietary education, by introducing an element of fun into eating, encouraging them to give audio feedback when consuming foods with different textures. 

Serious games (video games or interactive games] could introduce children to diverse foods, guiding them to what is good for them [25]. This could help surpass some neophobia and picky eating [26] in particular contexts. It also adds a performative aspect to behavior change, or an in-game transformation perspective, that is more usual in PA.

In pursuing innovative ways to support obesity prevention and being aware of the allure and promise of challenging games in health, this study took shape. It is a systematic review of the existent literature on the effect of serious games for promoting healthy diet behaviors (as well as PA) in children and adolescents. Data were also collected regarding the theories for change behind the game design, the (possible) acknowledgment of the kid’s environment, and performative aspects of the game (motion and tangibility). 

## 2. Materials and Methods

In order to conduct the present systematic review of the literature, a slightly modified PRISMA (Preferred Reporting Items for Systematic reviews and Meta-Analyses) checklist was followed [27]. 

The identification of papers was conducted through a search on PubMed, ACM Digital Library, Games for Health Journal, and IEEE Xplore with the following expression [(“serious game” OR gamification) AND (nutrition OR “health promotion”)]. 

The search was carried out in September 2022, where the filters were applied to exclude systematic reviews and meta-analyses and the inclusion of full papers, short papers, and extended abstracts. In total, publications were collected and analyzed, unless they were written in a language different from English, Portuguese, or Spanish.

After collecting all publications to be analyzed, three authors (SA, SL, and SC) reviewed the abstracts—each of them reads the abstract of the papers individually—to seek agreement between them in deciding which publications would be included. A third element (EP) participated in the discussion of included and excluded papers to solve disagreements in the decisions of the other two authors. 

The search retrieved duplicate articles, which were removed. The inclusion criteria were: papers evaluating an intervention in nutrition and/or PA, where there was a change in behaviors (for instance, increase in the intake of healthy foods/decrease in the intake of unhealthy foods or beverages; increase in PA; increase in knowledge about healthy behaviors) after the intervention. The exclusion criteria considered were: papers written in languages other than English, Portuguese, or Spanish (*n* = 3); narrative reviews (*n* = 70); studies not focused on nutrition and/or PA (*n* = 50); studies using patients as target population (*n* = 37); studies not using games, or where their role is only subsidiary (*n* = 35); studies which did not contemplate any intervention (*n* = 37); and studies not targeting children and/or adolescents (*n* = 57).

The revision of the abstracts excluded 289 studies from the 403 initially retrieved (after the exclusion of repeated abstracts), making a total of 95 articles to read in full. Next, the three authors that conducted the abstracts’ assessments and analyzed the full papers in detail opted for the exclusion of 83 of them. These exclusions were motivated by: a paper written in Spanish (*n* = 1); narrative reviews (*n* = 3); studies not focused on nutrition and/or PA (*n* = 7); studies using patients as target population (*n* = 6); studies not using games, or where their role is only subsidiary (*n* = 24); studies which did not contemplate any intervention (*n* = 19); and studies not targeting children and/or adolescents (*n* = 23).

**Figure 1:**
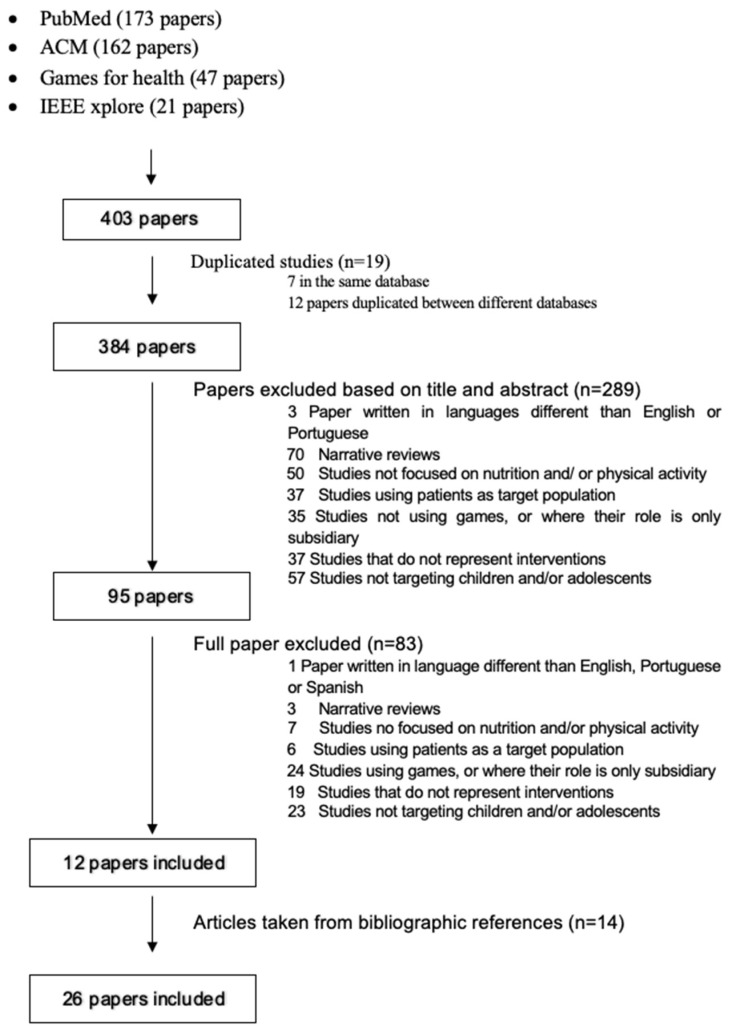
PRISMA diagram showing the screening process.

Only 12 papers met the inclusion criteria. Their reference lists were also reviewed, and another 14 studies were included in the revision. All these steps are represented in a flowchart (Figure 1).

The study information was systematized in a table including the following characteristics: first author’s name, year of publication, country of study, sample’s characteristics, area of intervention, type of study, description of the intervention, and finally, the results that each study had. The table is presented in Appendix A. 

## 3. Results

After using the predefined search strategy, 403 results were pulled from the databases. Three hundred and eighty-four remained for screening after duplications were eliminated. Following a review of the titles and abstracts, 95 papers were kept. After reading the studies, 12 papers fulfilled the inclusion criteria and were maintained for data extraction. An additional 14 pertinent papers were found and included in the final analysis by examining the references from the included reviews. The selection of 26 distinct studies for data analysis were totaled. Figure 1 displays the progression of the screening.

The 26 papers included in this revision corresponded to interventions conducted around the world between 2003 and 2022, the majority of them in the USA (*n* = 15), and the rest in the Netherlands (*n* = 1), Germany (*n* = 2), Italy (*n* = 2), UK (*n* = 1), Mexico (*n* = 2), Finland (*n* = 1), Australia (*n* = 1), and Canada (*n* = 1). In total, they represented 17 different games.

Half of the studies (*n* = 13) tested interventions directed both to healthy eating and PA, six studies focused merely on improving healthy eating, and two focused only on PA. The sample size ranged between 20 and 1578 participants; three of these studies included teams of parents and their offspring. When parents were involved, the primary objective was to receive information about the results of the intervention and to be able to assist with future behavioral change. The age of the participants mostly ranged between eight and fifteen years old, with only one of the studies including participants up to eighteen years old and another three children from three to eight years old.

Fifteen studies were randomized controlled trials, and eleven were quasi-experimental studies, with pre-and post-intervention evaluation. Regarding the period in which the intervention took place, it ranged from one day to three months.

Most of the intervention games (nine out of seventeen) were designed according to specific behavioral change theories, predominantly the social cognitive theory (SCT) [28] and the self-determination theory (SDT) [29], but also the Elaboration Likelihood Model [30]. Other behavioral theories were also used, such as the Transtheoretical Model of Change [31], Behavioral Inoculation Theory [32], Maintenance Theory [33], the Theory of Reasoned Action [34], Transportation Theory [35], and in two cases, Behavior Change procedures from the Michie inventory [36] were applied. Other theories were used from game design and theoretical education areas. Following the concept of serious gaming, one paper [37] reported using the theory of persuasive gaming and the positive gaming concept [38], and another one [39] reported using images from collaborative games in conjunction with parental PA modeling concepts. A third game [40] explored the idea of Situated and Embodied Cognition, including Gestural congruency. No theory was specified in the remaining games [41,42,43,44,45,46,47].

Serious games that did not use any particular behavioral theory aimed chiefly at increasing players’ nutrition knowledge or PA. Examples of nutrition information are knowledge about healthy/unhealthy foods, energy-dense foods, food quality vs. calorie content, or meal composition. As for the games with embedded behavior change theories, they expected some behavioral change in their players at the postgame (or follow-up), although with diverging goals: increase healthy food intake, fruits, and vegetables (FV) consumption, water intake, or level of PA; positive changes in anthropometric measures or attitudes and intentions; or to decrease consumption of sugary foods, sugar-sweetened beverages (SB), packaged snacks (PS), or non-healthy foods.

Regarding PA, serious games are a potent means to prompt movement and activity during gameplay. Of the ten games that included PA as an interventional area (alone or in conjunction with diet improvement), in five of them, motion control interfaces and movement sensors were used to imbed PA in the game (namely, Microsoft Kinect V2 sensor, SMALLab mixed reality platform, Fitbit activity trackers, or GPS mobile sensor). In the other six, motion does not occur in the game: they are uniquely cognitive-based (e.g., by delivering information on energy expenditure tied to caloric intake and healthy eating concepts). 

Games with nutrition as a unique interventional area (*n* = 7) are solely cognitive-based. As for the others, tangible elements (besides PC keyboards, touchscreens, and touchpads) are almost absent, except for one boardgame (that uses cards, dice, and pawns) and a mixed reality game that uses trackable wands for players to interact with the digital world (e.g., picking digital objects). Thus, natural foods are never present in these games. Nutrition communication is always cognitive-based.

Notwithstanding, in two serious games (Squire’s Quest! I and Squire’s Quest! II—two versions of the same game) players are elicited to eat FV during the day and to prepare authentic recipes with their parents (or other adults) by establishing specific goals in the game. During gameplay, they are taught how to make simple FV recipes (“Virtual Kitchen”) through brief demonstration video clips and then asked to set goals to make an actual recipe at home.

In five games, the children’s food environment is somehow acknowledged, ranging from simply referring to a grocer or restaurant and their food availability, to asking children to set solutions for environmental barriers to FV consumption and PA. 

Regarding the interventions’ impact assessment, this was generally carried out through questionnaires. Some studies, in addition to questionnaires, included anthropometric measurements, the determination of biochemical parameters, and the use of accelerometers to measure PA, as well as data collected during gameplay through in-game log files. One study performed a food-tasting test as a pretext to assess energy-dense food consumption post-intervention. Different measurements were used to assess eating behavior, including the Food Intake Recording Software System (FIRSSt)—a computer-based 24-h dietary recall program for children. Questionnaires were administered in person in most studies. However, five questionnaires were administered by phone to children or parents in cases where they were also the target of the study. One study [48] used the EGameFlow and guessed questionnaires to evaluate children’s enjoyment of the game and user experience satisfaction to link these concepts to learning. Besides that, Wengreen et al., 2021, estimated the daily FV consumption of all the children eating a school-prepared lunch using a waste-based measure by weighting and taking photos before/after lunch trays [44].

There was a control group in almost all experimental—sixteen out of seventeen—and four quasi-experimental studies. In some cases, the control group received written information; in others, lectures in a classroom context played generic games (such as web-based games) or participated in incomplete parts of the game. There were cases in which the control group constituted the wait list group and received the intervention sometime after the study.

The vast majority of interventions in nutrition had a positive impact, with changes in knowledge and behavior. Only one of the games [49] did not significantly impact the different variables tested. Several games increased children’s nutritional knowledge compared to the control or baseline group. Moreover, most serious games that embedded behavioral change theories registered positive results, despite the diversity of the foreseen goals. Globally, they reported an increase in the consumption of healthy foods or FV and a decrease in sugary or processed foods. Positive changes in intentions and attitudes toward healthy eating and physical exercise were also attained. In one study [48], enjoyment and user experience satisfaction with the game were positively correlated and significant predictors of learning.

Even so, some studies found effectiveness in both interventions (game and control), and others found significant positive results immediately after gameplay (and not in the follow-up). In one study [50], although players decreased the consumption of SB and PS, there were no positive effects in FV and water intake, PA, or screen time (or, generally speaking, a shift to a more balanced diet). The same happened in other studies, especially in variables such as the availability of FV at home or some objective variables (e.g., positive changes in anthropometric measurements—e.g., body mass index or waist measurement—or in fasting insulin (a biological parameter important for preventing diabetes).

None of the selected articles targeted for PA registered an increase in the PA after the game (reported by the kids or measured using an accelerometer); there were only positive changes in attitudes and intentions [39,51]. Solely one game, explicitly designed to increase PA [43], produced positive results during gameplay, with players achieving a speed representative of “moderate to vigorous activity”.

Lastly, eight of the seventeen games were designed following formative evaluations, and two included procedures to tailor content to the player’s preferences (e.g., FV was selected based on the child’s food preferences reported at baseline). Several games were beta-tested, and some were piloted before the final intervention. 

Formative evaluations used quantitative (e.g., surveys) and qualitative methods (e.g., focus groups, observation) to assess children’s interest in the game’s story (or what their preferred story genre was), or which characters were more appealing, but also, play issues (frequency of play, motives, and favorite games). Several followed iterative methodologies during game development, such as a user-centered design.

All these results are systematized in Table A1. 

## 4. Discussion

This study analyzes interventions using serious games to improve children’s and adolescents’ knowledge and practices of a healthy diet and PA.

The papers reviewed were published in peer-reviewed journals before September 2022 and scrutinized regarding the interventions’ characteristics and effectiveness and specific games’ characteristics/concrete features that push serious games away from solely cognitive forms of communication.

Our results confirm previous findings, which point to promising results in using serious games for health promotion and obesity prevention in terms of interventions in nutrition. The vast majority of interventions had a positive impact, with changes in knowledge and behavior. Only one game [49] did not significantly impact the tested variables. This is very positive, considering that in some cases the intervention was very brief and the players were only briefly exposed to the game (e.g., in one study, only 30 min–2 h/day, during one week). Therefore, we sought to find out if implementing some interventions over a more extended period of time could have a more significant impact.

In reality, as in other reviews [16], we found that the effects were relatively small. Moreover, there was also a need for long-term follow-up evaluations in most studies that could confirm if the results were sustainable. Our sample is very diverse in its goals, evaluation methods, and tools.

Unfortunately, the interventions targeted at PA were ineffective in the postgame. However, changes in attitudes and intentions were promising, but we were expecting positive changes in PA (besides PA knowledge) because nearly half of the serious games involved motor skills and some cognitive information, although this concurs with previous findings [18]. Some factors that could justify this result is the rather brief duration of the interventions, a higher resistance of less physically active players to the PA messages diffused in interventions [52], or the need to integrate the game intervention in a broader multi-component program [16].

Despite this drawback, we registered the positive influence of in-game PA, and in one study, it led the group to an average speed equaling “moderate to vigorous” aerobic activity levels [43]. This accords with previous research, which points to the effectiveness of active video games in supporting children’s PA and energy expenditure [53] (even if not reaching the daily PA recommendations) versus sedentary games more linked to children’s health education.

This output encourages the mentioned alternative approach of serious games in nutrition with a performative facet: that could foster sensory education by including natural healthy foods as tangible elements in the game, as opposed to solely cognitive-based games. The hypothesis is that effectiveness can be prompted by including taste/sensory activities in the game, such as having PA in active videogames. In the studies reviewed, real foods were never present in games, following the trend of serious educational games that revolve around cognitive skills [17]. Nutrition communication is always cognitive-based.

The foreseen perspective can be added to the extent of theories applied in serious games for nutrition and PA. In our review, most games included behavior change theories, such as the social cognitive theory or self-determination theory in their design, seeking to extend information to attitudes and behavior change. Moreover, other theories and concepts influenced by psychology were also used—namely, the idea of persuasive gaming or the positive gaming concept; concepts such as enjoyment and experience; or the Situated and Embodied Cognition. 

It is interesting to see that, alongside the demand for a more solid research base in design and evaluation [20], the research community is also concerned about exploring different theories in serious games. Embodied learning, for example, could be linked to sensorial education with foods, or self-efficacy [54] in researching and preparing foods. In this trend, there are already two games (Squire Quest! I and II) that prompt players to establish goals to make recipes at home, offering small cooking demonstration videos.

In our attempt to approach children’s reality in serious games, we have analyzed the (possible) use of a formative evaluation in the game/intervention design and the (possible) acknowledgment of the children’s food environment in the game narrative and concept.

Formative evaluations are crucial, helping to create the game/interventions for the target audience. Moreover, we found that twelve out of the eighteen games were designed following these evaluations using quantitative (e.g., surveys) and qualitative methods. Two also included tailoring mechanisms to suit the game to their players. Only a few games used formative evaluation to access nutritional or PA issues (versus play profiles, story, and character topics). In these examples, preferences, reasons, needs for nutritional information, or parental attitudes towards and support for their child’s PA were accessed. In one challenging particular game, recipes presented in a “Virtual Kitchen” were tested before their inclusion to ensure they were child-friendly, tasted good, and looked attractive [55].

Five of the reviewed serious games somehow recognize the children’s (occidental) food environment. However, there is a lack of games that expose the truth about the food industry and our social/eating environment. Some online games (not object to evaluation) [56] revolve their narrative around these problems as behavior change issues in the story [18] and as sparks of motivation. In our review, three games acknowledge these limitations and ask players to set solutions for environmental barriers to FV consumption and PA.

Finally, most serious games were targeted to 8–15-year-old children (as reflected in the interventions). This is a suitable age range as kids in this age group are progressively more autonomous in their food choices, making it essential that they are aware of options of what is good for their health and especially that they feel motivated to choose well. Additionally, it would be interesting to see more multiple-component interventions (vs. individual interventions) or simply interventions aimed at parents. As mentioned before, diet changes should be supported by the family and the family environment (and we only found two games that included parents and their offspring).

In our review, all the games were developed in Western countries (nine in the USA), which may indicate the regional prevalence of the obesity epidemic and the easy access to funding or technological means, while also setting the tone for the problems addressed in the games. It would be desirable to find studies in other parts of the world where children’s and adolescents’ eating habits and leisure time may differ.

Lastly, evaluation methods mostly comprised self-report (or parent-reported) questionnaires and interviews, or some objective measurements, such as the measurement of PA using accelerometers or biometrics—the latter generally not advancing positive results. Some qualitative methodologies could enrich these variables, bringing different indicators of efficacy, for example, through experiments evaluating whether kids positively respond to a food environment change (e.g., in school) in conjunction with a serious game intervention. Food-tasting tests could also be exciting tools to evaluate the game’s impact, especially if sensorial education is involved.

This study has some limitations that could interfere with the results. Its primary focus is nutrition, leaving some PA games and exergames out of the analysis. Even so, previous reviews confirm our results. The low number of scientific articles retrieved can indicate that this subject has yet to be fully explored, and more games should be developed with different theoretical perspectives and tested.

## 5. Conclusions

This review provides an overview of research interventions using serious games as strategies to promote healthy eating and PA in children. Despite some inconsistencies, our findings confirm that most studies in nutrition have been effective in increasing knowledge and/or changing kids’ diets to healthier ones. Notwithstanding PA, results seem to be mainly limited to the gameplay. The intrinsic entertainment and motivation of games, coupled with accurate information and a solid theoretical psychology ground, bring some hope for progress in obesity prevention.

All serious games analyzed, approached nutrition through cognition and only two versions of the same game elicited players to prepare recipes at home. A performative facet, using sensorial experiences with foods (seeking transformation in the course of the game), was never present. There is a need for more diversity of games because playing with foods is a good way for kids to get to know and appreciate novel foods, especially healthy ones with bitter tastes. 

Although formative evaluation is sometimes used in the game design, only a few examples gathered information about the player’s needs and behaviors about the intervention theme. Moreover, although some games acknowledged the children’s food environment and food choice pressures, these issues are mostly subtly referred to. From our point of view, serious games should pursue new perspectives other than the mainstream accountability of kids’ behaviors for the obesity epidemic. A broader and more ecological perspective is needed. 

Finally, as argued by other authors, interventions would benefit from being more research-based, possibly by having more prolonged intervention periods and more long-term evaluation follow-ups. To these claims, we add the benefit of exploring new theoretical grounds for serious games and possibly new evaluation methods to explore all the possibilities of communication in this area of research. 

## Data Availability

Not applicable.

## Appendix A

**Table A1:** Characteristics of the included studies.

Reference	Intervention Area	Description of the Intervention				Results	Theory Behind the Game	Performative Aspects			Acknowledgment of the Environment	Formative Evaluation or Tailoring
		Sample/Type of the Study	Duration and Intensity	Assessment Tools	Control			Application Domain	Interactional Elements	Contract with Real Foods		
Vepsäläinen et al., 2022. (Finland) [47] Mole’s Veggie Adventures app: a Mobile App to Increase Fruit and Vegetable Acceptance	N	3–6 y *n* = 221 Experimental	3–10 players; 4 sessions of which includes 6 FV each	Background questionnaire to parents (T0). Acceptability to try the FV presented during the game (questionnaire to parents) (T1)	CG had no play sessions with Mole’s Veggie Adventures app	Compared with the CG, the participants in the intervention arm had higher FV acceptance scores at follow-up 3–4 weeks after baseline.	No specific theory.	Cog	Mobile phone	No	No	No
Vlieger at al., 2022 (Australia) [45] VitaVillage: improving child nutrition knowledge	N	9–12 y *n* = 189 Experimental	2 sessions (~20 min)	Nutrition knowledge questionnaire (T0, T1)	CG played mathematics games	Compared to the control group, the intervention group’s overall nutrition knowledge was found to increase after playing Vita Village.	No specific theory.	Cog	Tablet	No	No	No
Wengreen et al., 2021 (USA) [44] FIT Game’s: Increase Fruit and Vegetable Consumption.	N	5–11 y *n* = 978 Experimental	32 episodes presented; 3 min each episode looped continuously throughout the lunch time	Daily FV consumption of all the children eating school-prepared lunch was estimated using a waste-based measure: before/after lunch-tray photos were taken and height (T0, T1, T2)—BMI and skin-carotenoid concentrations (T0, T1, T2)	CG had no play session with FIT Game	During the intervention phase, children attending the FIT Game schools consumed significantly more consumption FV compared to the baseline. This increase in at-school consumption was reflected in their skin carotenoid concentrations, which was also significantly different between the IG and CG.	No specific theory.	Cog	Television	Yes (game involves eating FV)	No	No
Frome et al., 2020 (Canada) [46] Foodbot Factory: a Mobile Serious Game to Increasing Nutrition Knowledge in Children	N	8–10 y *n* = 73 Experimental	10–15 min each day over a five-day period	Nutrition knowledge questionnaire (T0, T1). Nutrition knowledge for each of the four sub-scores measured with the validated Nutrition Attitudes and Knowledge (NAK) Questionnaire. BMI calculated based on parent-reported weight and height (T0)	CG played a control app called “My Salad Shop Bar”	Compared to the control group, children who used Foodbot Factory had significant increases in overall nutrition knowledge, and in Vegetables and Fruits, Protein Foods, and Whole Grain Foods sub-scores. No significant difference in knowledge was observed in the Drinks sub-score	No specific theory.	Cog	Android Tablet	No	No	No
Mack et al., 2020 (Germany) [42] Kids Obesity Prevention program (KOP): serious game for children with knowledge-based activities addressing the areas of nutrition, physical activity, and stress coping	N PA	9–12 y *n* = 82 Experimental	The IG played the game twice, over a 2-week period, with a different selection of game modules (45 min./session).	Knowledge questionnaire (T0, T1, T2). Changes in dietary behavior, Physical activity and Media consumption questionnaires (T0 + T2; child and parents reports) Acceptance of the game and Emotions during game play questionnaires (T1; child self-reports). Analysis of game data (Int) T2= 2 weeks after play	CG received a brochure with basic information about a healthy lifestyle—“How to make food fun” (food pyramid and PA).	Total knowledge increased with the game and remained at the 2-week follow-up. No positive changes were observed in PA.	No specific theory. Game extensively targets the Dietary Energy Density Principle (DED-P).	Cog Mot	Motion control interface (for moving the avatar in the game) + Tablet (choice selection)	No	No	No
Espinosa-Curiel et al., 2020a (Mexico) [57] FoodRateMaster: teaches the characteristics of healthy/unhealthy foods, bearing in mind children’s environment.	N PA	8–10 y *n* = 60 Quasi-experimental	12 game sessions of at least 15 min each, during 6 weeks (45 days)	Food knowledge and Food frequency questionnaires (T0, T1). Parent perception questionnaire (T1)	No control	Increased nutritional knowledge after gameplay. Increased frequency intake of two healthy foods, and decreased intake of 10 unhealthy foods. Positive influence in children’s attitudes (parent reported).	Game is grounded on the constructs of Behavioral Theory, Cognitive Theory and Social Cognitive Theory.	Cog Mot	Microsoft Kinect V2 sensor (basic physical movements in avoiding obstacles and for classifying food) + PC	Yes (6 levels that replicate real food establishments (e.g., a food truck, a restaurant, or a grocery store))	No	Interdisciplinary team and iterative methodology (user-centered design).
Espinosa-Curiel et al., 2020b (Mexico) [48] FoodRateMaster:				EGameFlow and GUESS questionnaires (T1)		Enjoyment and user experience satisfaction with the game were positively correlated and significant predictors of learning.						
Holzmann et al., 2019 (Germany) [37] Fit, Food, Fun (FFF): a serious game with European country-specific food items, to increase food knowledge and PA	N PA	12–14 y *n* = 83 Quasi-experimental	IG played the FFF game individually for 3 consecutive days, 15 min/session.	Dietary behavior, physical activity, and healthy eating attitudes accessed by questionnaire (T0). Nutritional knowledge questionnaire (T0, T1)	CG received a teaching intervention, performed in a classic lecture format (with similar content).	Total knowledge increased in both groups, especially in the CG.	Game of the NUDGE platform. Influenced by the concept of serious gaming, the theory of persuasive gaming, and the concept of positive gaming.	Cog	Tablet	No	No	Pre-game design survey, conducted with 300 adolescents. On preferences, motives, and needs regarding nutritional information and digital gameplay. Usability tests and focus groups.
Baranowski et al., 2019b (USA) [49] Escape from Diab and Nanoswarm: Invasion from Inner Space: 2 video games designed to lower risks of type 2 diabetes and obesity by changing youth diet and physical activity behaviors.	N PA	10–12 y *n* = 145 (Children in the 85th—99th %ile of BMI) Experimental	Each game had 9 sessions (each episode/session lasting 60 min). Treatment group played Diab and Nano in sequence (total of 2 to 3 months)	BMI assessment; parent self-reported data (T0). Fasting insulin assessment; 32-item Fruits and Vegetables Food Frequency Questionnaire (FV-FFQ); 22- item sweetened beverage FFQ (T0, T1). PA assessed using accelerometer (≤7 days). Gameplay data collected over the Internet (Int) T2 = 2 months after play	CG was a wait list group that received the intervention at the end of the 5-month postbaseline assessment.	No significant differences were detected in any of the tested outcome variables.	Several theories, including Social Cognitive Theory, Self-Determination Theory, Behavioral Inoculation and Transportation Theories and the Elaboration Likelihood Model.	Cog	Computer	No	Yes (barriers to attain diet and physical activity goals were included in “Escape from Diab”)	Quantitative and qualitative methods applied to examine child preferences for storyline genres and plot content of nonviolent video games; also, computer access, knowledge, and game-play frequency. Tailoring: goal-settings were selected according to the player’s behaviors and preferences reported at the baseline.
Baranowski et al., 2011 (USA) [58] Escape from Diab and Nanoswarm: Invasion from Inner Space		10–12 y *n* = 145 (Children in the 85th—99th %ile of BMI)		3 nonconsecutive days of 24 h dietary recalls. 5 consecutive days of physical activity using accelerometers. Self-reported height, weight, waist circumference and triceps skinfold assessments (T0, P1, P2, P3). P1 = immediately after Diab P2 = immediately after Nano P3 = 2 months after intervention.	CG received 2 kits (booklet+ CD), comprising a knowledge-based nutrition game and 8 sessions of web-based online games (related to diet, PA and obesity), with questions after each session.	Significant increase in FV intake, in the IG when compared with the CG, including 5 months later. No positive changes in water intake, physical activity, or body composition.						
		Experimental										
Hermans et al., 2018 (Nederlands) [59] Alien Health Game (AHG): videogame designed to teach elementary school children about nutrition and healthy food choices, while engaging in short cardio exercises. (Dutch version)	N PA	10–13 y *n* = 108 Experimental	30-min long-play sessions, on 2 consecutive days at children’s school.	BMI assessment (T0) Nutrition Knowledge questionnaire (T0, T1, T2) Food-taste test (T1, T2) T2 = 2-week after play.	CG played a web-based nutrition game for the same period of time (Super Shopper).	No substantial differences between IG and CG. IG revealed better knowledge only shortly after playing the game (vs. CG). No evidence for behavior change.	“Mixed reality” platform (AHG uses both digital components as well as tangible, physical components). Theoretically grounded in Situated and Embodied Cognition, including Gestural congruency, and also Behavior Change procedures from Michie inventory.	Cog Mot	SMALLab (mixed-reality educational platform, that uses motion-tracking cameras mounted in a ceiling or on a trussing system, and trackable wands) + interactive whiteboard.	No	No	No
Johnson-Glenberg et al., 2014 [40] (USA)		10–13 y *n* = 20	50 min of play and play-observing (9 min of actual play/dyad).	Nutrition Knowledge questionnaire (T0, T1)	CG: the same performative food choices at the interactive whiteboard, without a game narrative or cardio exercises.	Total knowledge increased in both groups after intervention, but IG outperformed the CG (especially in the follow-up).						
Alien Health Game (AHG)		Experimental										
Johnson-Glenberg et al., 2013 (USA) [60] Alien Health Game (AHG)		9- 10 y *n* = 19 Quase-experimental	45 min of play and play-observing		No Control	Knowledge regarding food choices increased significantly. Evidence of knowledge transfer of general nutrition principles.						

Thompson et al., 2017 (USA) [55] Squire’s Quest! II (SQ2): 10-episode online serious videogame promoting FV intake to preadolescent children, by testing the effect of implementation intentions on FV goal attainment and consumption.	N	9–11 y *n* = 400 parent/child dyads Experimental Four groups: Action; Coping; Action+ Coping; Control. Action plans state “how” a goal will be achieved. Coping plans identify a potential barrier and corresponding solution.	10-episode, online videogame; each session/episode lasting about 25 min; up to three months to play all 10 episodes.	Telephone interviews with children (T1). Game-play data (INT)	Control condition did not create implementation intentions. Parents were involved through newsletters and a dedicated website.	Overall, mean goal attainment was high (FV consumption goals and recipe goals), with no statistically significant differences between groups. Children were more likely to select the F recipe. Program satisfaction was high.	SQ2 is theoretically grounded in SCT, SDT, Behavioral Inoculation Theory, Maintenance Theory and Elaboration Likelihood Model.	Cog	Computer	Yes (brief demonstration videoclips taught players how to make simple FV recipes (‘Virtual Kitchen’)Players set goals to make a recipe at home)	Yes (children selected barrier-specific solutions to potential obstacles to FV intake)	Extensive formative work conducted to ensure children understood the behavioral procedures and could complete them, and also that the game was appealing (e.g., interviews, alpha testing, beta testing). Recipes tested to ensure they were child-friendly, tasted good, and looked attractive. Pilot study to test procedures (enrollment, data collection, intervention delivery) and as final beta test of the online game.
DeSmet et al., 2017 (USA) [61] Squire’s Quest! II (SQ2)				Asking behavior and home FV availability questionnaires, (child-reported and parent-reported, respectively) (T0, T1, T2). T2 = 3 months after play.		Despite increasing (and decreasing, at follow-up), children asking behaviors did not lead to an increase in FV at home or to more FV consumption.						
Cullen et al., 2016 (USA) [62] Squire’s Quest! II (SQ2)				24 h dietary recalls conducted via phone (3 unannounced 24 h dietary recalls, FV intake averaged) (T0, T2). T2 = 3 months after play.		Action and coping groups participants reported higher V intake at dinner. Significant increases over time of F intake at breakfast, lunch, and snack (at follow-up).						
Thompson et al., 2015 (USA) [63] Squire’s Quest! II (SQ2)		9–11 y *n* = 387 parent/child dyads Experimental (Four groups)		24 h dietary recalls conducted via phone (3 unannounced 24 h dietary recalls, food and beverage intake averaged) (T0, T1, T2). Demographic data (T0) T2 = 3 months after play.		Children who only created action plans showed increased intake of FVs at follow-up, and favorable changes in energy density and key nutrients. F intake increased over time, regardless individual IG results.						

Sharma et al., 2015 (USA) [51] Quest to Lava Mountain (QTLM): an action-adventure game that requires children to make simulated appropriate food choices and stay physically active to move steadily through the game and succeed.	N PA	9–11 y *n* = 94 Quase-experimental	90 min/week for 6 weeks recommended	Demographic data (T0). Two random 24 h dietary recall interviews. Child self-report surveys to assess Diet and Physical Activity Habits and Related Psychosocial Mediators; anthropometric measures (T0, T1) QTLM usability, exposure to, and progress achieved (INT)	CG were children in three comparison schools (wait list to play QTLM)	Children in IG reported decreased sugar consumption and higher N/PA attitudes pre- to post intervention. No significant effects on PA.	Social Cognitive Theory and the Theory of Reasoned Action	Cog	Computer	No	No	No
Marchetti et al., 2015 (Italy) [64] Gustavo in Gnam’s Planet: a game to improve knowledge on healthy foods and increase their consumption.	N	14–18 y *n* = 83 Qusai-experimental	30 min–2 h/day, during one week.	Healthy food knowledge questionnaire, Food frequency questionnaire, and Interest questionnaire (T0, T1)	No control	Knowledge on healthy diet improved with the game. Increased consumption of different healthy foods and lower consumption of sugary snacks post-play.	Theoretically grounded in the Transtheoretical Model of Change, the SCT, the SDT, and the Elaboration Likelihood Model.	Cog	Computer	No	No	No
Saksono et al., 2015 (USA) [39] Spaceship Launch (SL): game with Fitbit activity trackers, to increase parent and child PA.	PA	3–8 y *n* = 14 (and 15 caregivers) Quase-experimental	≥once a week total of 3 weeks	Demographics, PA intention and parental PA modeling and support (T0). PA intention survey. Experience with the game and impact in intentions and modeling behavior (semi-structured interviews with parents) (T1).	No control	Children and parents reported an increase in the intention to be physically more active.	Informed by SCT, SDT, and concepts of Parental PA modeling (as predictor of children’s PA) and collaborative games in family	Mot	Fitbit activity trackers + Web app (home devices and large interactive display in gym sessions).	No	No	Formative and post-gameplay studies, to guide the game design. Focus groups with parents to probe parental attitudes towards, and support for, their child’s PA.
Majumdar et al., 2013 (USA) [50] Creature-101: online game to promote energy balance-related behaviors (EBRBs)	N PA	11–13 y *n* = 590 Quasi-experimental	7 sessions, 30 min each, for 1 month	Frequency and amount of the targeted behaviors (Eat-Move instrument) and demographic data (T0, T1)	CG played 8 minigames (Whyville). matching standard science/health curriculums of NYC. Nutrition games excluded and networking activities deactivated.	Decrease in the consumption of SB and PS. No significant positive effects in FV and water intake, PA, or screen time.	Based on SCT and SDT. Behavioral change procedures based on Michie inventory. Embedded in a social networking structure (Elgg).	Cog	PC (game experience mostly through a MMO virtual world (Tween)	No	No	Focus groups and in-class observations to obtain feed-back about game structure and if it met behavior change objectives (link of the game to real-life behaviors). Beta-testing.
Macvean et al., 2012 (UK) [43] iFitQuest: Location-aware mobile exergame, played on the iPhone, by using Google Maps. The game is made up of a number of “mini-games”, each designed to target a different type of fitness.	PA	12–15 y *n* = 25 Experimental	3 h session, with 30 min of play (15-min/mini game)	Background questionnaire (T0). Exertion and enjoyment of exercise with the game assessed through questionnaire; interview of expert PA teacher (that observed sessions) (T1). In-game log-files and rating collected during play (INT)	No control	IG achieved levels of speed representing “moderate to vigorous” activity, while playing iFitQuest.	No specific theory. Being physically demanding, iFitQuest grounds itself in published exergames design requirements to maintain motivation and enjoyment.	Mot	iPhone (exercise of game-players in real world physical movements are used to control the virtual character).	No	No	Preliminary school-based field study to guide the game development
Amaro et al., 2006 (Italy) [4] Kalédo: a board-game to teach nutrition knowledge and to influence dietary behavior, regarding Mediterranean diet.	N PA	11–14 y *n* = 241 Experimental	2 to 4 players; 24 play sessions (15–30-min-long play sessions, once a week)	Nutrition knowledge, dietary intake, and physical activity questionnaires (T0, T1). BMI measurement (T0)	CG had no play sessions with Kalèdo.	Significant increase in nutrition knowledge and in weekly vegetable intake (vs. CG).	Unspecified. Combined purpose of promoting/discouraging specific dietary behaviors.	Cog	Board Game (cards, paws, play pieces, dice, “kaleidoscopes”)	No	No	No
Cullen et al., 2005 (USA) [65] Squire’s Quest! I: a 10-session interactive game to enable children to increase FJV intake, through activities promoting increasing asking behaviors and increasing skills in FJV preparation through virtual recipes.	N	8–12 y *n* = 1578 Experimental	25 min/session, in a total of 10 sessions/episodes, for 5 weeks.	4 days of dietary intake assessment using FIRSSt, (T0, T1). Demographic data (T0).	GC without intervention	IG increased the consumption of fruit and 100% juice at snacks and the consumption of regular vegetables at lunch.	Based on social cognitive theory, the game used multiple exposure and fun in educational activities to increase preferences for and FJV consumption.	Cog	Computer	Yes (game involves students preparing FJV recipes in a virtual kitchen, and then setting goals to make recipes at home).	Yes (game involves a problem-solving routine to help players think of practices to increase the likelihood of eating more FJV).	Focus group discussions with fourth-grade children to assess interest in the story line and to identify child-desired characters’ characteristics. Tailoring: FJV were selected based on the child’s food preferences reported at baseline.
Baranowski et al., 2003 (USA) [66] Squire’s Quest! I						IG increased their FJV consumption by 1.0 servings more than the CG.						

CG—Control Group; Cog—Cognitive skills; FJV—Fruit, 100% fruit Juice and non-fried Vegetables; FV—Fruit and vegetable GUESS—Game User Experience Satisfaction Scale; IG—Intervention Group; INT—During Intervention; MMO—Massively Multiplayer Online game; Mot—Motor skills; N—Nutrition; NA—Not Applicable; NYC—New York City; PA—Physical activity; PC—Personal computer; PS—Packaged Snacks; SB—Sweetened Beverages; SCT—Social Cognitive Theory; SDT—Self-Determination Theory; T0—Intervention baseline; T1—Immediately post-game; T2—a few months post-game; V—Vegetable.
